# Individual Adaptation in Cross-Country Skiing Based on Tracking during Training Conditions

**DOI:** 10.3390/sports7090211

**Published:** 2019-09-12

**Authors:** Stefan Adrian Martin, Roxana Maria Hadmaș

**Affiliations:** 1 Physiology Department, University of Medicine Pharmacy Science and Technology Târgu Mureș, Târgu Mureș 540139, Romania; 2 Community Nutrition and Food Hygiene Department, University of Medicine Pharmacy Science and Technology Târgu Mureș, Târgu Mureș 540139, Romania

**Keywords:** elite athlete, training, sport performance

## Abstract

Research on heart rate (HR), mean arterial pressure (MAP) and blood pressure (BP) during specific training stages is less common in endurance athletes, whereas resting BP and HR are less studied in relationship to HR_max_. In the current study, the objective was to conduct a medium-term HR, BP and MAP analysis while tracking individual training outcomes. The study was conducted during the 2017–2018 season, over 43 days and 1033 km of training volume, on 12 competitive male cross-country ski athletes. One VO_2max_ test was performed 10 days before the start of the training program. After the test, training volume and intensity was preset for each subject, according to the general training methodology. Early morning HR, MAP and BP measurements were taken as part of the basic functional analysis. Training volume was correlated to both distance (*p* = 0.01, r = 0.85, CI95% = 0.80 to 0.88) and training HR%, namely the percentage of HR_max_ (*p* = 0.01, r = −0.47, CI95% = −0.58 to −0.34). Both the supine (sHR) and orthostatic HR (oHR) values were significantly correlated with the training intensity. We obtained a significant correlation between sHR and oHR values and the training objective (*p* = 0.01). An increased oHR was correlated to high intensity training activity (HIT) during the second training session (*p* = 0.01). Heart rate and blood pressure measurements represent predictive functional adaptation parameters over different training phases. We highlight a link between sHR, oHR, MAP data, and the athletes’ ability to perform in lower effort zones during physical exertion. However, we failed to validate MAP as a cardiovascular stress indicator following high intensity training.

## 1. Introduction

Planning the training intensity is of particular importance in sports performance, while monitoring the athlete can offer specific data regarding the adaptation process, the recovery status or the main physiological changes that occur. Some of the most common measurements used for training monitoring are the heart rate (HR), blood pressure (BP) and mean arterial pressure (MAP), along with blood lactate and sleep quality.

Day by day training activity may significantly differ from laboratory research conditions [1,2,3]. As a result, field-based research can be more relevant to daily training activity, even if common methods are used in the study methodology. Over the training stages, cardiovascular and neural adaptations seem to be related to various training characteristics, such as effort intensity, effort volume and environmental conditions, during either short or long training periods. Of the main changes, physical effort influences vascular remodeling [4]. Yet, aerobic physical training favors angiogenesis, positively influencing the number of capillaries and therefore the gas exchange area, while improving oxygen diffusion and vagal tonus [5,6]. Without proper adaptation, the heart rate will increase due to reduced parasympathetic and increased sympathetic activity [7]. Increased long-term sympathetic activity induces cardiovascular stress, a drop in muscular strength, agility and an increased reaction time, influencing both resting and effort cardiovascular parameters.

Despite the use of HR as a predictor of autonomic cardiovascular activity, few data are available regarding heart rate and blood pressure during rest-to-effort and effort-to-rest transitions [8]. During the resting or physical effort period, neural adaptation will strongly influence the HR measurement result and therefore the muscle contraction frequency changes the hearts sympathetic stimulation. During high intensity training (HIT), performances can change due to a drop in both cardiac output and HR_max_, of which values fail to exceed a 90% peak. Both power and muscle contraction frequency can describe the effort intensity better than the HR value, which can easily be affected by other factors [9,10]. During physical exertion, both systemic vasoconstriction and local vasodilatation fail to bring a significant change in diastolic blood pressure, whereas systolic blood pressure reaches a plateau near maximal exercise intensity [11,12]. The main outcome is an increased MAP, which can be maintained during the post effort period [13]. Contrary to HR_max_ use during training, several authors have utilized HR measurements during recovery periods [14,15,16]. Research papers on resting HR and BP tend to be less specific in elite cross-country skiing during medium-term periods, while long-term MAP and HR are less studied in relation to HR_max_.

A small number of research papers that investigate long-term training performance following HIT activity are available [13,17,18]. Yet, HR use during short-term periods is well known. Several groups are using HR data for individual training and performance tracking. However, less data regarding the use of resting BP and MAP in training is being published. Performance drop is related much more to training-induced fatigue and reduced muscle fiber recruitment. Starting from this point, our objective was to conduct a medium term HR, BP and MAP analysis while tracking individual training performances. Based on our hypothesis, specific physiological HR, BP and MAP changes can be associated with individual adaption according to the training characteristics. By testing such a hypothesis, daily activity during both general and specific training conditions can be significantly improved by using simple, cost efficient and noninvasive methods.

## 2. Materials and Methods

We conducted an observational study between October and December 2017, during part of the 2017–2018 cross-country ski training season. To conduct the study and publish the current results, written informed consent was received from: (1) the participants, (2) federal management, and (3) the University Ethical Committee.

### 2.1. Participants

The study group consisted of 12 international competitive male cross-country skiers with a mean age of 23 (18–28). To be included in the study group, the subjects had to fulfil the following criteria: (1) male cross-country ski athlete, (2) general medical acceptance, (3) >18 years old, (4) currently competing at professional national or international level. Exclusion was pre-set by using the following criteria: (1) medical incompatibility with the pre-determined training program, (2) health condition that would inhibit the study activity, (3) age less than 18 or (4) lack of international or national competitive activity.

Two individuals (n = 2) were excluded at the start of the study due to age (<18 years old).

### 2.2. Procedures

The study was conducted during the 2017–2018 season, over 43 days and 1033 km of training volume, on a sample of 12 competitive male cross-country ski athletes. Each athlete undertook one VO_2max_ test 10 days before the start of the training program, which was preset for each subject according to the general training methodology. Five (n = 5) training zones were determined for each athlete (45–100% of VO_2max_), namely: training zone 1: zone 1 (Z1, 45–65% of VO_2max_), training zone 2: zone 2 (Z2, 66–80% of VO_2max_), training zone 3: zone 3 (Z3, 81–87% of VO_2max_), training zone 4: zone 4 (Z4, 88–93% of VO_2max_) and training zone 5: zone 5 (Z5, 94–100% of VO_2max_) [19]. Morning heart rate (HR) and blood pressure (BP) were measured daily as part of the basic functional analysis over the pre-determined training period.

### 2.3. Maximum Rate of Oxygen Consumption during the Incremental Exercise Test (VO_2max_)

Each athlete performed the VO_2max_ test by undertaking the Bruce Maximal Testing Protocol [20]. The test was applied using Cosmed Quark CPET equipment (Rome, Italy) and a Cosmos T150 running ergometer over seven effort stages (1 to 7), each consisting of 3 min lengths. The VO_2max_ test was conducted after calibrating the Cosmed unit with known O_2_ (16%) and CO_2_ (4%) concentrations. The flow meter was calibrated at the start of each test using the Cosmed Syringe (3 L). The VO_2max_ measurement was validated by applying the following criteria: respiratory exchange ratio (RER) >1.10, ±10 b/min of the predicted HR_max_ and/or ≤150 mL O_2_/min changes.

From the VO_2max_ test data, the following parameters were of particular importance: maximum oxygen consumption (VO_2max_), maximum reached heart rate (HR_max_), the ventilatory threshold 1 (VT_1_, b/min) and the ventilatory threshold 2 (VT_2_, b/min). The ventilator thresholds were determined by applying the *V Slope* method [21].

### 2.4. Training Monitoring

The training analysis was completed using the global positioning systems (GPS): Polar V800 (+/–2% error) (Kempele, Finland) and a Polar H7 Bluetooth HR monitor (Kempele, Finland). The following parameters were monitored during training: (1) heart rate (b/min, %), (2) distance (km), (3) movement speed (km/h), (4) time (min), (5) positive (Dif +) and (6) negative altitude gain (Dif −).

### 2.5. Training Periodization

Two training sessions (n = 2) were run each day during 33 of the 43 study days (76.74%). On each day that had two training sessions (T_1_ and T_2_), T_1_ was programed in the morning, whereas T_2_ was programed after a 6 to 8 h recovery period. Against the mean training volume (1033 km), a difference between 5 and 10% (51.6–103.3 km) was monitored between the study subjects.

Fifty four (n = 54) training sessions were conducted on skis, eleven sessions (n = 11) involved trail running, and five sessions (n = 5) were conducted on roller skis.

### 2.6. Basic Functional Analysis

Heart rate (HR) and blood pressure (BP) measurements were included in the study methodology as the basic functional analysis. Each measurement took place in the early morning. Heart rate (HR, b/min) and blood pressure (BP, mmHg) analyses were performed by applying an orthostatic test using the following methodology: 3 min supine positioning followed by HR measurement (sHR, b/min); 3 min orthostatic positioning followed by HR measurement (oHR, b/min). In addition to HR analysis, blood pressure (BP) was measured using an aneroid sphygmomanometer (mmHg) under the following protocol: 3 min supine positioning, followed by BP measurement (sSBP, mmHg, systolic blood pressure; sDBP, mmHg, diastolic blood pressure); 3 min orthostatic positioning followed by orthostatic BP measurement (systolic blood pressure, oSBP mmHg; diastolic blood pressure, oSBP, mmHg). At least two (n = 2) measurements were taken each time, resulting in a mean value if the first measurement did not exceed ±5 mmHg. If such a difference was obtained, a third measurement was conducted. Each measurement was repeated if the sounds were not clearly defined [22].

### 2.7. Secondary Measurements

By using ΔTA (difference between systolic blood pressure and mmHg) and ΔP (difference between heart rate values, b/min), the Crampton Index (CI) was calculated for each data set. Later, MAP (mean arterial pressure) was obtained by applying the following formula:

Equation (1). Formula used to calculate (a) the Crampton Index and (b) MAP:

(1) $$\begin{array}{ll} a. & \mathrm{Crampton}\;\mathrm{Index}\;=\;25\;\times \;(3.15\;+\;\frac{\Delta \mathrm{TA}}{10}\;-\;\frac{\Delta \mathrm{TP}}{20})\\ b. & \mathrm{MAP}\;=\;\frac{\left(2\;\times \;\mathrm{DBP}\right)\;+\;\mathrm{SBP}}{3} \end{array}$$

The following criteria were used to interpret the Crampton Index: <50 (insufficient adaptation), 50–75 (poor adaptation), 75–100 (good adaptation), >100 (very good adaptation). The MAP normal range was considered to be between 70 and 110 mmHg [23,24].

### 2.8. Analysis

GraphPad Prism 5.0 software (GraphPad Software Inc., Sand Diego, CA, USA) was used for the statistical analysis. The main statistical indicators used to describe the sample were the mean and median values. The D’Agostino and Pearson normality test was applied for data normalization. The Wilcoxon matched pairs test was used to compare the evolution of one parameter, while through the Spearman test we calculated the correlation between two parameters. A two-tailed Mann–Whitney test was applied to identify the difference between two items. The significance level was set at α = 0.05, with a pre-determined confidence interval of 95% (CI95%).

## 3. Results

### 3.1. Effort Capacity

Individual effort capacity was assessed by performing incremental exercise testing. VO_2peak_ reached a mean value of 78.86 mL/min/kg (74.6–82.3). VT_1_ was determined at 75.52% (73.2–78.9%), while VT_2_ was determined at 86.5% of HR_max_ (84.4–88.1%).

### 3.2. Training Effort Analysis

The physical training activity consisted of 1.033 km and 4.7432 min, respecting the general training guidance described in Table 1 for both the T_1_ and T_2_ training seasons. Of the training volume, 81.7% of the activity (843.961 km) was performed between 45–80% of VO_2max_, equivalent to the low aerobic effort training zone. Differences were monitored between T_1_ and T_2_ regarding the volume, specifically through effort time (minutes of effort), and distance (kilometers), along with altitude gain (meters) and mean effort HR (b/min) (Table 1).

### 3.3. Basic Functional Evaluation Data

The mean sHR value was determined at 54.2 b/min, while oHR reached 81.3 b/min (*p* = 0.01). The Crampton Index was calculated at 101.5 of the mean value, while both the sSBP and sDBP values (112.8 and 67.2 mmHg, respectively) were lower compared to the oSBP and oDBP measurements (117.6 and 79.6 mmHg, respectively) (*p* = 0.0017).

**Figure 1:**
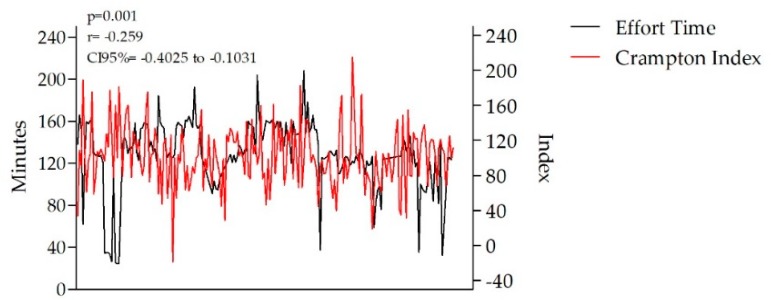
Correlation between effort time and the Crampton Index (*p* = 0.001).

Training volume was correlated to both distance (*p* = 0.01, r = 0.85, CI95% = 0.80 to 0.88) and training HR%, namely the % from HR_max_ (*p* = 0.01, r = −0.47, CI95% = −0.58 to −0.34). Both the sHR and oHR values were significantly correlated with the training performed 12 h before the basic functional analysis. As a result, we obtained a statistically significant correlation between the analyzed sHR and oHR values and the training objective (*p* = 0.01). Based on the data, the athletes’ capacity to perform and maintain lower effort HR ranges was significantly correlated with both their sHR and oHR values (*p* = 0.01, r = −0.38, CI95% = −0.50 to −0.24), whereas T_1_ and T_2_ volume was significantly correlated with the Crampton Index (*p* = 0.001) (Figure 1).

The T_1_ results were correlated with the Crampton Index, unlike the T_2_ training data. The measurements are illustrated in Table 2 for both training activities.

The individual ability to perform over a predetermined HR range was correlated to training intensity, as shown through the specific training zones illustrated in Table 3. An inappropriate HR% range (>10%), contrary to the training objective, was correlated with increased sHR–oHR and sBP–oBP values (*p* = 0.0116, r = −0.21, CI95% = −0.38 to −0.04) during basic functional measurements.

Resting sHR and oHr were correlated with both sBP values (*p* = 0.0026, r = 0.21, CI95% = 0.07 to 0.35) and exercise HR (*p* = 0.0001, r = 0.293, CI95% = 0.149 to 0.424). No statistically significant findings *(p* > 0.05) were obtained between both sBP–oBP measurements and T_1_ volume (*p* = 0.553). Further, MAP failed to be correlated with T_1_ volume and training intensity (*p* > 0.05), except during high aerobic training (*p* = 0.007, r = −0.199, CI95% = −0.34 to −0.05). Yet, the SBP–DBP data was correlated with the effort HR (*p* = 0.0065, r = 0.23, CI95% = 0.06 to 0.39), while supine SBP–DBP results were correlated with mean effort HR (*p* = 0.0059, r = 0.23, CI95% = 0.06 to 0.39), as shown in Table 4 for SBP and Table 5 for DBP.

## 4. Discussion

In this study we chose to monitor the major physiological changes that occur during several training phases, including the low intensity–high volume stage and the high intensity–low volume stage. Starting from our hypothesis, the resting MAP, BP and HR values describe individual training-induced adaptations. Related articles have used HR and BP data to describe non-effort cardiovascular and nervous system recovery. However, a different approach was proposed when applying the current methodology.

Among the most common used physiological parameters, MAP, BP and HR are easy to monitor. In our results, HR, BP and MAP were correlated with the physical effort HR. A decrease in cardiovascular control, and possibly in power through muscle strength, may be associated with muscle fiber recruitment and neural adaptation. We believe, but did not demonstrate through the current methodology, that muscle fiber activation, overall fatigue and general physical stress induced the main changes in BP and HR, similar to the findings of Jing-Jing Wan et al. (2017) [25].

### 4.1. Physiological Induced Changes

Cardiac activity is defined as a repetitive electric wave measured through the number of contractions per time unit [26]. As described by Imai et al. (1994) [27], heart rate analysis during training can be used to evaluate vagal tone restoration, while continuous HR recovery is used to describe both parasympathetic and sympathetic activity [28].

Starting from the recovery stage and the individual effort capacity, HIT activity can induce changes in resting HR and BP values, similar to those found by Grace et al. (2018) [29]. By performing aerobic training, both the parasympathetic nerves and the stroke volume will improve resting HR values [30], while an enhanced peripheral vessel resistance can reduce BP over a short-term period [31]. According to Mont et al. (2009) [32], bradycardia is a parasympathetic-mediated adaptation, which was undefined in our results due to the training stage and the training volume. Based on our outcomes, both HR and BP measurements were linked to the subjects’ capacity to perform in the predetermined effort zones during several training stages.

### 4.2. Resting Heart Rate Measurement and Training

An advanced heart rate kinetic indicates training-induced sensitivity during various stages, as shown both in our results and in the outcomes from Nelson et al. (2014) [33]. Based on our findings, resting heart rate is significantly correlated with daily physical training intensity. During T_1_, aerobic training induced an increase in resting sHR, whereas both anaerobic and low aerobic training intensity were associated with lower resting heart rate values. This outcome is different to early findings regarding post-exercise HR [34]. Neural adaptations can induce changes in resting HR values. Therefore, the mechano-receptors could have caused changes in both muscle length and tension over T_2_ due to T_1_ training-induced fatigue. However, several papers have confirmed that aerobic physical exercise will drop resting HR values through parasympathetic tone [35].

From a general perspective, athlete adaptation can be influenced by both the volume and the physical effort intensity, as seen in oHR due to several hormonal- and neural-induced changes. Anaerobic and low aerobic training were related to a drop in oHR, while an increased oHR value was encountered when aerobic and high aerobic physical training was undertaken (T_1_). This outcome could be related to training-induced vascular adaptations, among which an increased blood flow could be obtained during both physical effort and resting periods [4]. During T_2_, low aerobic, aerobic and high aerobic activities were correlated with a drop in oHR, whereas anaerobic physical effort conducted during T_2_ was related to a higher oHR. This finding was similar to that of other papers, which confirmed the effect of aerobic physical effort on resting HR [36]. However, many other factors can also be involved, including the recovery process.

No statistically significant correlations (*p* > 0.05) were obtained between the high aerobic training zone and resting HR. Resting sHR was higher during T_2_ when related to aerobic, high aerobic and anaerobic physical training, whereas in T_1_, a drop in resting sHR was correlated with low aerobic training. As published by Oh et al. (2003) [37] and Fagard et al. (2003) [38], resting heart rate varies according to the training period. Therefore, early physical training conducted above VT_1_ was related to low sHR, while the same effort intensity conducted during T_2_ had the opposite effect, as confirmed in Table 2 and Table 3.

Increasing resting HR affected the individuals HR response during physical exercise, which is contrary to Bellenger et al. (2016) [39]. They confirmed that a slight but constant increase in HR may indicate training-induced fatigue, as seen in T_2_ compared to T_1_ over sHR unlike oHR. According to Thomson et al. (2016) [40], an increased resting heart rate may be associated with fatigue. However, our results seem to confirm the athletes’ ability to perform at high intensity based on HR control. A short distance HIT activity held during T_2_ was significantly correlated with increased HR and BP values 12 h post effort, similar to the findings of Guerra et al. (2014) [41], which illustrated similar changes in HR and no concerns in the BP analysis.

### 4.3. Blood Pressure and Mean Arterial Pressure Resting Measurements and Training

Following the study outcomes, a 4–10 mmHg increase in resting BP values was found as a result of aerobic training (81–87% of VO_2max_), unlike Chobanian et al. (2003) [42], who described a 4–9 mmHg drop during low intensity high volume training. Hanssen et al. (2017) [43] published a similar increase in resting blood pressure due to high intensity training (≥88% of VO_2max_). They concluded that improper volume and intensity staging can be identified early by analyzing HR and BP data during a 24 h period. Only a few papers have found similar outcomes during short periods of BP monitoring.

Bo-Ae Lee et al. (2016) [44] concluded that aerobic exercise induces a drop in both sBP and oBP, whereas post physical exercise values can be similar. Contrary to the main findings in the literature, sSBP failed to correlate with T_1_ training intensity. Aerobic intensity during T_1_ physical training was significantly correlated with oSBP, as Kelley et al. (2001) found [45]. Following a linear intensity guide, few changes were observed in SBP, while both DBP and MAP values remained the same or slightly dropped post physical exercise. Unlike Cornelissen et al. (2005) [46], low aerobic training intensity was correlated with an increased oSBP, whereas both aerobic and high aerobic training intensity were correlated with low measured values. The current study outcomes were different for T_2_ training, in that sSBP dropped if low aerobic training was performed, whereas aerobic physical effort induced an increased value. No statistically significant results were obtained in terms of oSBP values and both high intensity and low intensity effort during T_2_ training, while MAP values were not related to the time of physical exertion but to high aerobic training intensity. A possible explanation for this could be related to vascular modulation, increased vasodilatation capacity, and several changes in autonomic tone, which may help in reducing vascular resistance. Such an outcome could also be related to an improved vascular diameter, as seen in athletes compared to inactive subjects [47]. MAP was statistically correlated with physical exercise HR and aerobic volume, but no data were obtained showing a correlation between high aerobic or anaerobic training intensity and MAP values.

Our papers does confirm other recent findings in the literature regarding blood pressure and athlete performance [34,48].

### 4.4. Study Limitations

Lack of heart rate variability (HRV) monitoring is a limitation of the current research, while non-specific VO_2max_ testing could have limited the current VO_2max_ values. However, day by day training analysis in elite athletes is an important strength of the current study methodology as we applied specific training conditions, unlike previous research [2,49,50].

In future, we will propose a similar hypothesis but with several methodology changes. Among them, we would seek to conduct an advanced HR analysis by using: (1) HRV analysis, (2) continuous BP, (3) HR acceleration, and (4) HR recovery, in order to fully assess individual adaptation.

## 5. Conclusions

Heart rate and blood pressure measurements represent predictive functional adaptation parameters over different training phases. Based on our outcomes, we have highlighted a link between sHR, oHR, MAP, and athletes’ ability to perform at lower physical effort intensities. An increased resting HR value was related to an elevated physical effort HR, without any reported pace or intensity changes. However, oHR was significantly increased during T_2_ due to reduced recovery time between the first (day 1) and the second assessment (day 2), limiting HR_max_ during HIT training. Further, oHR was associated with HIT training during T_2_, whereas MAP was not validated as a possible cardiovascular stress indicator following high intensity training.

Further data is needed to understand the performance drop, along with the HR_max_ limitation over maximal effort, and its relationship with both the training stage and the individual effort capacity.

## Figures and Tables

**Table 1:** General data indicating training (T_1_–T_2_) outcome.

Information Related to One Individual Training Session	Mean Value (Min to Max)		Statistical Data between T_1_ and T_2_	
	T_1_	T_2_	*p*	*r*
Distance, km	28.23	10.83	0.51	0.05
	(9.22–52.26)	(2.09–22.55)		
Time, minute	129.3	63.66	0.01	0.31
	(24.17–208)	(15.52–130.5)		
Positive altitude gain, m	430.5	232.5	0.007	0.26
	(35–1115)	(10–965)		
Negative altitude gain, m	390	162.7	0.01	0.24
	(47.78–94)	(0–650)		
HR %	69.75	65.56	0.0014	0.19
	(59–100)	(55–92.13)		
Z1, %	4.9 (0–78)	2.2 (0–85)	0.06	0.15
Z2, %	6.1 (0–88)	6.2 (0–86)	0.01	0.37
Z3, %	4.1 (0–77)	5.9 (0–76)	0.01	–0.31
Z4, %	26.4 (0–87)	18.6 (0–91)	0.02	–0.30
Z5, %	58.5 (0–100)	67.1 (0–100)	0.046	0.16

Note: T_1_—first training of the day; T_2_—second training of the day; *p*—probability level; r—Pearson product-moment correlation coefficient; HR—heart rate; VO_2_ = maximum rate of oxygen consumption; VT_1_ = ventilator threshold 1, VT_2_ = ventilator threshold 2, Z5 = anaerobic power training zone, Z4 = anaerobic training zone, Z3 = high aerobic training zone, Z2 = aerobic training zone, Z1 = low aerobic training zone.

**Table 2:** Comparative data for the T1 and T2 training activities and the Crampton Index.

Crampton Index Mean Value	Training Data Mean Value		Statistical Result			
			*p*	r	CI95%	
					Upper	Lower
101.5	T_1_ training data					
	Distance, km	28.23 (9.22–52.26)	0.008	−0.19	−0.33	−0.04
	Time, minute	129.3 (24.17–208)	0.07	−0.13	−0.27	0.01
	Pace, km/h	14.15 (8.5–25.2)	0.33	−0.07	−0.22	0.07
	Positive altitude gain, m	430.5 (35–1115)	0.012	−0.20	−0.35	−0.03
	Negative altitude gain, m	390 (47.78–94)	0.02	−0.18	−0.34	−0.02
	HR %	69.75 (59–100)	0.0007	0.30	0.15	0.43
	Z1, %	4.9 (0–78)	0.6687	−0.03	−0.18	0.11
	Z2, %	6.1 (0–88)	0.6863	−0.03	−0.18	0.12
	Z3, %	4.1 (0–77)	0.0626	0.13	−0.01	0.2
	Z4, %	26.4 (0–87)	0.0014	0.23	0.08	0.37
	Z5, %	58.5 (0–100)	0.0009	−0.24	−0.38	−0.09
	T_2_ training data					
	Distance, km	10.83 (2.09–22.55)	0.9654	0.00	−0.16	0.16
	Time, minute	63.66 (15.52–130.5)	0.6278	−0.03	−0.20	0.12
	Pace, km/h	11.28 (7.5–51.5)	0.0667	−0.16	−0.33	0.01
	Positive altitude gain, m	232.5 (10–965)	0.1819	−0.13	−0.31	0.06
	Negative altitude gain, m	162.7 (0–650)	0.4968	–0.06	–0.25	0.13
	HR, %	65.56 (55–92.13)	0.0438	0.16	–0.00	0.32
	Z1, %	2.2 (0–85)	0.8572	0.01	–0.15	0.18
	Z2, %	6.2 (0–86)	0.7330	–0.02	–0.19	0.13
	Z3, %	5.9 (0–76)	0.2283	–0.09	–0.26	0.06
	Z4, %	18.6 (0–91)	0.7851	–0.02	–0.18	0.14
	Z5, %	67.1 (0–100)	0.5423	–0.05	–0.21	0.11

Note: T_1_—first training of the day; T_2_—second training of the day; *p*—probability level; r—Pearson product-moment correlation coefficient; HR—heart rate; VO_2_ = maximum rate of oxygen consumption; VT_1_ = ventilator threshold 1, VT_2_ = ventilator threshold 2, Z5 = anaerobic power training zone, Z4 = anaerobic training zone, Z3 = high aerobic training zone, Z2 = aerobic training zone, Z1 = low aerobic training zone.

**Table 3:** sHR–oHR statistical analysis over training intensity (Z1–Z5).

Parameter 1 Mean Value		Parameter 2 Mean Value		sHR and Parameter 2 Analysis Results				oHR and Parameter 2 Analysis Results			
sHR, b/min	oHR, b/min	Effort Intensity		*p*	r	CI95%		*p*	r	CI95%	
						Upper	Lower			Upper	Lower
54.25	81.34	T_1_ training data									
		Z5, %	4.9	0.015	−0.18	−0.32	−0.03	0.012	−0.18	−0.33	−0.03
		Z4, %	6.1	0.0282	−0.08	−0.23	0.07	0.034	−0.15	−0.30	−0.00
		Z3, %	4.1	0.247	0.08	−0.06	0.23	0.135	0.11	−0.03	0.26
		Z2, %	26.4	0.001	0.3	0.15	0.43	0.001	0.42	0.29	0.54
		Z1, %	58	0.001	−0.23	−0.37	−0.08	0.004	0.262	−0.39	−0.11
		T_2_ training data									
		Z5, %	2.2	0.023	0.18	0.02	0.34	0.002	0.24	0.08	0.39
		Z4, %	6.2	0.012	0.20	0.04	0.36	0.001	0.33	0.17	0.47
		Z3, %	5.9	0.046	0.16	−0.002	0.32	0.005	−0.28	0.12	0.43
		Z2, %	18.6	0.044	0.16	−0.00	0.32	0.011	0.21	0.04	0.36
		Z1, %	67.1	0.007	−0.22	−0.37	−0.05	0.003	−0.29	−0.44	−0.13

Note: T_1_—first training of the day; T_2_—second training of the day; *p*—probability level; r—Pearson product-moment correlation coefficient; CI95%—confidence interval of 95%; sHR—supine heart rate; oHR—orthostatic heart rate, Z5 = anaerobic power training zone, Z4 = anaerobic training zone, Z3 = high aerobic training zone, Z2 = aerobic training zone, Z1 = low aerobic training zone.

**Table 4:** sSBP–oSBP statistical analysis of effort training intensities (Z1–Z5).

Analysed Parameter				Results							
Parameter 1 Mean Value		Parameter 2 Mean Value		sSBP and Parameter 2 Analysis Results				oSBP and Parameter 2 Analysis Results			
sSBP, mmHg	oSBP, mmHg	Training Zones over T_1_–T_2_ Trainings		*p*	r	CI95%		*p*	r	CI95%	
						Upper	Lower			Upper	Lower
112.8	117.6	T_1_									
		Z5, %	4.9	0.067	0.06	−0.09	0.22	0.0832	0.01	−0.14	0.17
		Z4, %	6.1	0.588	0.04	−0.11	0.20	0.540	−0.04	−0.20	0.11
		Z3, %	4.1	0.397	−0.67	−0.22	0.09	0.006	−0.21	−0.36	−0.05
		Z2, %	26.4	0.452	−0.06	−0.21	0.10	0.026	−0.17	−0.32	−0.01
		Z1, %	58	0.553	−0.04	−0.20	0.114	0.003	0.23	0.07	0.37
		T_2_									
		Z5, %	2.2	0.731	0.03	−0.14	0.20	0.445	0.06	−0.11	0.24
		Z4, %	6.2	0.244	0.10	−0.07	0.27	0.593	0.04	−0.13	0.22
		Z3, %	5.9	0.162	0.12	−0.05	0.29	0.299	0.09	−0.08	0.26
		Z2, %	18.6	0.008	0.22	0.05	0.38	0.703	0.03	−0.14	0.20
		Z1, %	67.1	0.009	–0.22	−0.38	−0.05	0.315	–0.08	−0.25	0.08

Note: T_1_—first training of the day; T_2_—second training of the day; *p*—probability level; r—Pearson product-moment correlation coefficient; CI95%—confidence interval of 95%; sHR—supine heart rate; oHR—orthostatic heart rate, Z5 = anaerobic power training zone, Z4 = anaerobic training zone, Z3 = high aerobic training zone, Z2 = aerobic training zone, Z1 = low aerobic training zone.

**Table 5:** sDBP–oDBP statistical analysis of effort training intensities (Z1–Z5).

Analyzed Parameter				Results							
Parameter 1 Mean Value		Parameter 2 Mean Value		sDBP with Parameter 2 Analysis Results				oDBP with Parameter 2 Analysis Results			
sDBP, mmHg	oDBP, mmHg	Training Zones over T_1_–T_2_ Trainings		*p*	r	CI95%		*p*	r	CI95%	
						Upper	Lower			Upper	Lower
67	79	T_1_									
		Z5, %	4.9	0.1986	0.10	−0.05	0.25	0.3240	0.07	−0.08	0.23
		Z4, %	6.1	0.1949	0.10	−0.05	0.25	0.5450	0.04	−0.11	0.20
		Z3, %	4.1	0.6372	−0.12	−0.12	0.19	0.0933	−0.13	−0.28	0.02
		Z2, %	26.4	0.1858	−010	−0.26	0.05	0.1432	−0.11	−0.27	0.04
		Z1, %	58	0.8644	−0.01	−0.17	0.14	0.1617	0.11	−0.04	0.26
		T_2_									
		Z5, %	2.2	0.5652	−0.05	−0.22	0.12	0.1266	−0.13	−0.30	0.04
		Z4, %	6.2	0.8553	0.01	−0.15	0.18	0.6851	−0.03	−0.20	0.14
		Z3, %	5.9	0.6263	−0.04	−0.21	0.13	0.4646	0.06	−0.11	0.23
		Z2, %	18.6	0.7171	−0.03	−0.20	0.14	0.1837	0.11	−0.06	0.28
		Z1, %	67.1	0.5209	0.05	−0.11	0.22	0.8292	−0.01	−0.19	0.15

Note: T_1_—first training of the day; T_2_—second training of the day; *p*—probability level; r—Pearson product-moment correlation coefficient; CI95%—confidence interval of 95%, sSBP—supine systolic blood pressure; oSBP—orthostatic systolic blood pressure heart rate; Z5—anaerobic power training zone; Z4—anaerobic training zone, Z3—high aerobic training zone, Z2—low aerobic training zone, Z1—warm up zone.
